# Improving the Flexibility and Water Resistance of Thermo-Compressed Guar Gum Films by Blending Natural Rubber for Use in Sustainable Packaging Applications

**DOI:** 10.3390/polym18080956

**Published:** 2026-04-14

**Authors:** Prasong Srihanam, Nuanchai Khotsaeng, Yodthong Baimark

**Affiliations:** 1 Biodegradable Polymers Research Unit, Department of Chemistry and Centre of Excellence for Innovation in Chemistry, Faculty of Science, Mahasarakham University, Maha Sarakham 44150, Thailand; 2 Faculty of Science and Health Technology, Kalasin University, Namon District, Kalasin 46230, Thailand; nuanchai.k@ksu.ac.th

**Keywords:** guar gum, natural rubber, composite film, mechanical properties, water resistance

## Abstract

Guar gum (GG), a typical biopolymer, has found widespread use in packaging applications due to its biodegradability, non-toxicity, and low price. However, the further application of GG is significantly limited by its poor flexibility and water resistance. In this study, GG/natural rubber (NR) films were prepared by thermo-compressing hand-kneaded pastes made from GG powder and fresh NR latex. Various NR contents—5, 10, 20, and 40 wt%—were investigated. Water-resistant properties were determined by moisture absorption, water dissolution, surface wettability, and water vapor permeability. Fourier transform infrared spectroscopy indicated interactions between the dispersed NR phases and the GG matrix. Scanning electron microscopy revealed distinct phase separation between the GG and NR phases in the films. All GG/NR films exhibited excellent interfacial adhesion between GG and NR phases. Tensile results indicated that an increase in the amount of NR in the GG-based films led to a decrease in both maximum tensile strength and Young’s modulus, while elongation at break increased. GG/40% NR films exhibited an elongation at break of 17.5%, which is a substantial increase of 415% compared to pure GG films. The addition of NR showed improved water-resistant properties of GG-based films; however, the rate of biodegradation during soil burial decreased as the NR ratios increased. These thermo-compressed GG/NR blends hold promise as sustainable alternatives to single-use plastic packaging applications.

## 1. Introduction

Conventional plastic packaging, derived from fossil fuel resources, offers significant convenience for packaging a variety of products. This convenience has contributed to a steady increase in the use of plastic packaging, particularly for single-use packaging. It is widely recognized that non-biodegradable fossil-based polymers, which constitute plastic packaging waste, require hundreds of years to decompose. This prolonged degradation process leads to significant environmental pollution in both oceans and landfills [1]. The goal of ongoing research and development is to create environmentally friendly biodegradable polymers to replace conventional plastic packaging. Bio-based polymers, designed to have a lower carbon footprint than fossil-based polymers, are the focus of this particular effort [2].

Polysaccharides, such as starch, cellulose, chitosan, alginate, and guar gum (GG), demonstrate remarkable biodegradability and film-forming properties, thereby attracting considerable research interest for their prospective applications as biodegradable and sustainable materials [1,3]. Nonetheless, these polysaccharides possess specific limitations that must be resolved for efficient packaging applications, including the necessity for appropriate melt processing, increased flexibility, and enhanced water resistance. The majority of these reports concentrate on the melt processing of thermoplastic starch [4]. Research on the melting process of alternative polysaccharides remains limited, which could lead to a wider and more diverse application of polysaccharide-based packaging. Some applications reported include the melt processing of thermoplastic alginate [5,6,7], thermoplastic guar gum [8], and thermoplastic chitosan [9,10,11].

Guar gum (GG) is a polysaccharide that is prepared from guar beans and is non-toxic, inexpensive, and readily available [12]. It presents considerable opportunities for research and development in environmentally friendly and sustainable packaging solutions. GG has been thoroughly researched and utilized across multiple domains, including biomedical applications [12,13], the food sector [14], wastewater treatment [14,15], cosmetics [14], the petroleum industry [16], and packaging solutions [17,18,19]. However, GG products are typically produced using GG aqueous solutions, which require a significant amount of solvent and take a considerable time to evaporate. This process poses limitations on industrial production. Melt processing enables the continuous industrial production of GG products, allowing for a wider variety of shapes. To our knowledge, only a few reports discuss the formation of GG-based films using melt processing techniques [8,20]. By adding glycerol, GG films became more flexible but had a lower water resistance [20].

Polysaccharide film also has a significant disadvantage, namely, it has low flexibility due to the large number of hydrogen bonds. To improve the flexibility of polysaccharide-based films, different non-volatile plasticizers, such as glycerol [21,22] and low-molecular-weight poly(ethylene glycol) [23,24], are used. However, these small-molecule plasticizers may migrate during storage, which can lead to a reduction in the films’ flexibility [25,26]. Another significant drawback of polysaccharide films is their high sensitivity to water, which restricts their use in packaging applications due to moisture exposure from both the surrounding air and the products being packaged [27,28].

Natural rubber (NR) is identified as *cis*-1,4-polyisoprene. It is found in fresh latex from *Hevea brasiliensis* trees, comprising approximately 25–50 wt% by weight [29]. NR also includes non-rubber components such as proteins, carbohydrates, lipids, and minerals [30]. NR is a bio-based polymer known for its high flexibility, attributed to its low glass transition temperature of approximately −65 °C [31]. The use of NR blends with different polymers has been shown to enhance flexibility [32,33,34,35] and improve water resistance [36,37]. The compatibility of the continuous polymer matrix with the dispersed NR phases is a key factor that affects the mechanical properties of polymer blends [32,33,34,35]. Incorporating NR will reduce the flexibility of polymer blends if they have low interfacial adhesion. This work hypothesizes that fresh NR latex contains proteins and phospholipids that act as phase compatibilizers, increasing the flexibility of GG films when blended with NR. Furthermore, the strong hydrophobicity of NR is expected to enhance the water-resistant properties of GG-based films.

The objective of this study was to prepare GG/NR blend films using a thermo-compression technique. GG powder was hand-mixed with fresh NR latex to make a paste. The paste was kneaded and rolled with a rolling pin. The paste was then scissors-cut into pellets. A compression molding machine formed pellets into films. The study investigated the effect of the NR ratio on chemical structures, thermal properties, crystalline structures, phase morphology, mechanical properties, moisture absorption, water dissolution, surface wettability, water vapor permeability, film opacity and color, and biodegradation in the soil burial of the GG/NR films compared to GG and NR films.

## 2. Materials and Methods

### 2.1. Materials

Food-grade guar gum (GG) powder, containing a minimum of 82 wt% galactomannan and exhibiting a viscosity of 6000 cps in a 1% *w*/*v* aqueous solution, was sourced from Chanjao Longevity Co., Ltd. (Bangkok, Thailand). Fresh natural rubber (NR) latex with a total solid content (TSC) of 49 wt% and a dry rubber content (DRC) of 35 wt% was obtained from *Hevea brasiliensis* trees of the RRIM 600 clone in Kalasin province, Thailand. The latex was stabilized by adding 2.5 vol% of a 28 wt% ammonium hydroxide solution.

### 2.2. Preparation of Thermo-Compressed GG/NR Films

**Figure 1:**
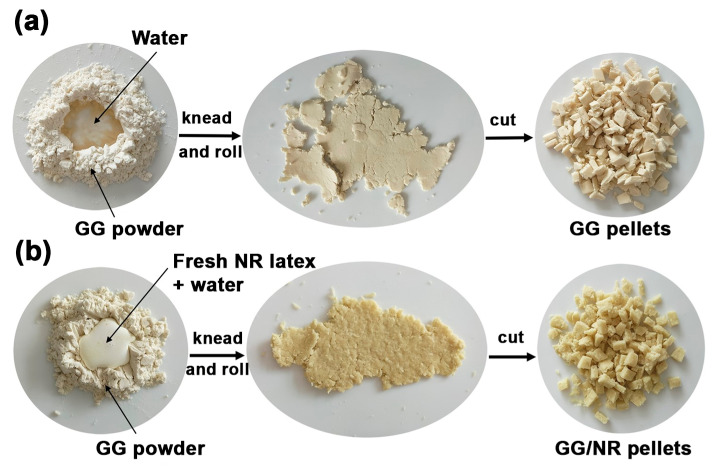
Preparation of (**a**) GG and (**b**) GG/NR pellets.

Pure GG pellets were produced by kneading and rolling 10 g of GG powder with 30 g of distilled water. The resulting paste-like mixture was then cut into pellets using scissors, as shown in Figure 1a. For GG/NR pellets, GG powder was mixed with diluted fresh NR latex before the kneading and rolling process. The total water content in all formulations was set at three times the weight of the GG, based on the 49 wt% TSC of the fresh NR latex. The blends were prepared with GG/NR ratios of 95/5, 90/10, 80/20, and 60/40 %*w*/*w*, calculated using the 35 wt% DRC of the fresh NR latex. The paste-like GG/NR mixtures were also cut into pellets using scissors, as depicted in Figure 1b. The blending formulations for all the mixtures are summarized in Table 1.

The film samples were prepared through thermo-compression at 120 °C for 5 min under a compression force of 5 MPa, utilizing a compression molding machine (Auto CH Carver, Wabash, IN, USA). The resulting film samples were dried in an airflow oven at 30 °C for 4 h. For comparison, NR film was prepared using the film casting method. A fresh NR latex (10 g) was dried in an air oven at 70 °C for 72 h. All film samples were stored at ambient temperature (25–30 °C) and relative humidity (50–60%) for 14 days prior to characterization [38].

### 2.3. Characterization of Thermo-Compressed GG/NR Films

#### 2.3.1. Fourier Transform Infrared Spectroscopy

The chemical structures of the film samples were analyzed with a Fourier transform infrared (ATR-FTIR) spectrometer (INVENIO-S, Bruker, Karlsruhe, Germany) equipped with an attenuated total reflection (ATR) diamond. The ATR-FTIR spectra were recorded in a wavenumber range of 500 to 4000 cm^−1^, an accumulation of 32 scans, and a resolution of 4 cm^−1^.

#### 2.3.2. Thermogravimetric Analysis

The thermal decomposition properties of the film samples were analyzed with a thermogravimetric analyzer (TGA, SDT Q600, TA-Instruments, New Castle, DE, USA) under nitrogen flow at a rate of 100 mL·min^−1^. A heating rate was 20 °C·min^−1^.

#### 2.3.3. X-Ray Diffraction Analysis

The crystalline structures of the film samples were determined with an X-ray diffractometer (XRD, D8 Advance, Bruker, Karlsruhe, Germany). A CuKα source was operated at 40 kV and 40 mA. A scan rate was 3° min^−1^.

#### 2.3.4. Scanning Electron Microscopy

The phase morphology of the film samples was determined with a scanning electron microscope (SEM, TM4000Plus, Hitachi, Tokyo, Japan) at 15 kV. The film samples were cryo-fractured in liquid nitrogen and were sputter-coated with gold before analysis.

#### 2.3.5. Tensile Test

The tensile properties of the film samples were evaluated with a universal testing machine (LY-1066B, Dongguan Liyi Environmental Technology Co., Ltd., Dongguan, China) with a 100 kg load cell at 25 °C and 50% RH. Tensile testing samples were 60 mm × 10 mm rectangular strips. An initial gauge length was set at 40 mm, and a crosshead speed was set at 50 mm.min^−1^. Five films for each film sample were tested, and their mean values were reported.

#### 2.3.6. Moisture Absorption Test

The film samples (20 × 20 mm) were dried in an air oven at 105 °C for 24 h before weighing (W_1_). The film samples were stored in a desiccator with 75% RH. At the interval time, the film samples were weighed (W_2_). The moisture absorption of the film samples was calculated using the following equation.

Moisture absorption (%) = [(W_2_ − W_1_)/W_1_] × 100 (1)

#### 2.3.7. Water Dissolution Test

The film samples (20 mm × 20 mm) were dried in an air oven at 105 °C for 24 h before weighing (W_3_). The film samples were then immersed in 50 mL of distilled water under shaking at 100 rpm at 25 °C for 24 h. The film samples were then dried in an air oven at 105 °C for 24 h before weighing (W_4_). The water dissolution of the film samples was calculated using the following equation.

Water dissolution (%) = [(W_3_ − W_4_)/W_3_] × 100 (2)

#### 2.3.8. Water Contact Angle Determination

The surface wettability of the film samples was assessed using the sessile drop method with a contact angle analyzer (OCA11, DataPhysics Instruments, Filderstadt, Germany). The water contact angle on the film surface was recorded at 15 s after a 2.5 µL water droplet was placed, measuring both the left and right contact angles, which were then averaged. For each film sample, three separate measurements were conducted and averaged.

#### 2.3.9. Water Vapor Permeability

The water vapor permeability (WVP) of the film samples was determined using the desiccant method. A bottle containing 10 g of anhydrous calcium chloride was sealed with the film (452.16 mm^2^) and weighed. The sealed bottle was then placed in a desiccator at 25 °C, maintained at 75% RH using a saturated calcium chloride solution. The weight of the sealed bottle was recorded every 24 h. The slope of the weight versus time curve was analyzed using linear regression. The WVP value was calculated using the following equation [39]. Data was collected in triplicate.

WVP (g·mm^−1^·h^−1^·Pa^−1^) = (Slope × d)/(A × ΔP) (3)

where d is the thickness of the film sample (mm), A is the film area (mm^2^), and ΔP is the vapor pressure difference in the film (1753.55 Pa) [40].

#### 2.3.10. Film Thickness and Opacity Measurement

The film thickness of the film samples was measured using a digital micrometer (Mitutoyo, Tokyo, Japan) with an accuracy of 0.001 mm. The film opacity of the film samples was determined by absorbance at a wavelength of 600 nm (A_600_) using a UV-Vis spectrophotometer (Cary 60, Agilent Technologies, Victoria, Australia). The film opacity was calculated using the following equation [41].

Film opacity (mm^−1^) = A_600_/X (4)

where X is the thickness of the film sample (mm).

#### 2.3.11. Film Color

The color of the film samples was measured using a color spectrophotometer (UltraScan Vis, HunterLab, Reston, VA, USA) with a D65/10° illuminant observer. The CIELAB color space measurements included lightness (L*), red-green (a*), and yellow-blue (b*).

#### 2.3.12. Biodegradation in Soil

The biodegradation test of the film samples was conducted by burying them in soil. The soil used was of a type commonly employed for planting trees. The film samples were cut into pieces, each measuring 15 mm by 15 mm, dried at 50 °C for 24 h, weighed (W_i_), and then placed inside nylon mesh bags with a mesh size of 1 mm, followed by the soil burial test. Three replicates of each sample were placed at a depth of 5 cm in the soil. Distilled water was applied every other day. The pH value was maintained between 6.0 and 7.0, while the moisture level was kept within a range of 50% to 60%. The films were collected every 2 days over a period of 8 days. They were then dried at 50 °C for 24 h. Afterwards, any soil residue on the nylon mesh bags was gently brushed off before being weighed (W_f_). Weight loss of film samples was calculated from Equation (5). Data was averaged in triplicate. Thereafter, the remaining film was removed from the nylon mesh bags and photographed.

Weight loss (%) = [(W_i_ − W_f_)/W_i_] × 100 (5)

### 2.4. Statistical Analysis

The analysis of the experimental data was conducted using one-way ANOVA, subsequently applying Duncan’s post hoc test with SPSS version 22.0. The findings are presented as mean ± standard deviation (SD), indicating statistically significant differences at *p* < 0.05.

## 3. Results and Discussion

### 3.1. FTIR Analysis

**Figure 2:**
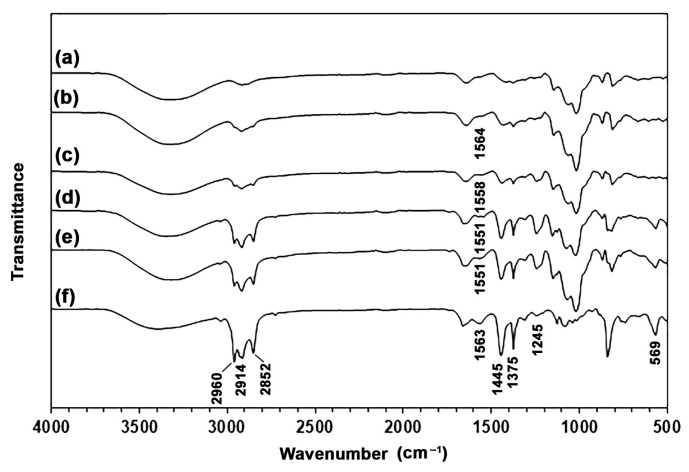
ATR-FTIR spectra of GG/NR films with GG/NR ratios of (a) 100/0, (b) 95/5, (c) 90/10, (d) 80/20, (e) 60/40, and (f) 0/100% *w*/*w*.

The chemical structures of the film samples were analyzed using ATR-FTIR, with the results presented in Figure 2. The pure GG films displayed the vibration bands corresponding to O–H stretching at 3326 cm^−1^, C–H stretching at 2916 cm^−1^ [22,42], ring stretching of GG, and O–H bending associated with water at 1644 cm^−1^ [43,44]. Additionally, CH_2_ bending was observed at 1414 cm^−1^, along with C–O–C stretching at 1146 cm^−1^ and glycosidic bond stretching at 812 cm^−1^ [42,45].

The NR films exhibited vibration bands characteristic of *cis*-1,4-polyisoprene, including CH_3_ stretching at 2960 cm^−1^, CH_2_ stretching at 2914 and 2852 cm^−1^, –C=C– stretching at 1662 cm^−1^, CH_2_ bending at 1445 cm^−1^, CH_3_ bending at 1375 cm^−1^, symmetric C–O–C stretching and CH_2_ twisting at 1245 cm^−1^, =C–H wagging at 842 cm^−1^, and in-plane bending of C–C–C at 569 cm^−1^ [46]. The vibration bands corresponding to N–H stretching at 3389 cm^−1^, along with N–H bending and C–N stretching related to protein (amide II) at 1563 cm^−1^, were also observed. This presence is presumably attributed to the protein characteristics found in fresh NR latex [30].

In GG/NR films, bands corresponding to the functional groups of NR were distinctly identified, including CH_3_ stretching (2960 cm^−1^), CH_2_ stretching (2914 and 2852 cm^−1^), protein (amide II) (1551–1567 cm^−1^), CH_2_ bending (1445 cm^−1^), CH_3_ bending (1375 cm^−1^), twisting CH_2_ (1245 cm^−1^), and in-plane bending C–C–C (569 cm^−1^). The intensity of these bands increased significantly with increasing NR ratios, indicating that GG/NR films with varying NR contents could be successfully prepared. The wavenumbers of amide II bands from fresh NR latex were measured at 1564, 1558, 1551, and 1551 cm^−1^ for GG/NR films with 5%, 10%, 20%, and 40% NR, respectively. The shift to lower wavenumber in the amide II bands shows that proteins from fresh NR latex are interacting with the GG film matrix [47,48].

### 3.2. Thermogravimetric Analysis

**Figure 3:**
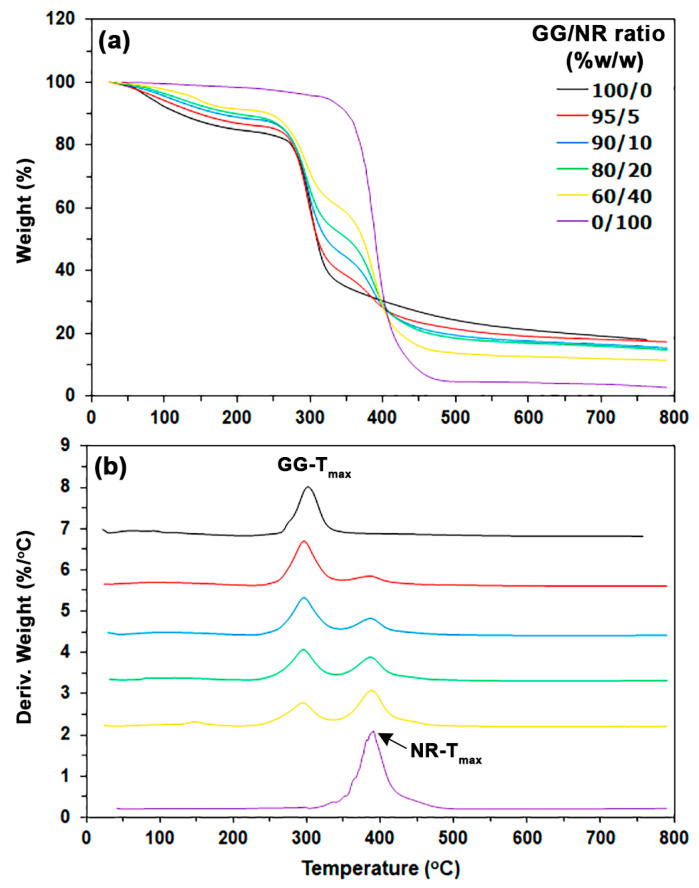
(**a**) TG and (**b**) DTG thermograms of GG/NR films with varying GG/NR ratios.

Figure 3 presents thermogravimetric (TG) and derivative TG (DTG) thermograms for the film samples. Table 2 outlines the results from the TG and DTG analyses. The pure GG films exhibited two distinct stages of weight loss: the first stage was attributed to moisture evaporation occurring between 50 °C and 150 °C, while the second stage was associated with the cleavage of galactose and mannose units from the GG chains, which took place between 250 °C and 400 °C [22,49]. The pure GG films exhibited decomposition temperatures of 81 °C at 5% weight loss (T_5%_), 121 °C at 10% weight loss (T_10%_), and 309 °C at 50% weight loss (T_50%_), while the char residue measured at 800 °C was 18.05%. The maximum decomposition temperature of the GG, referred to as GG-T_max_, was determined to be 302 °C based on the DTG thermogram analysis.

The NR films showed one stage of weight loss between 300 °C and 500 °C. The T_5%_ value was noted at 322 °C, the T_10%_ value at 351 °C, and the T_50%_ value at 389 °C, with a char residue of 2.73% remaining at 800 °C. The NR films demonstrated higher T_5%_ and T_10%_ values compared to the pure GG films, indicating that NR films have lower moisture content than pure GG films. This finding may be explained by the fact that NR is more hydrophobic than GG. It is well known that GG is hydrophilic due to its structure as a polysaccharide sugar, which contains many hydroxyl groups that can form strong hydrogen bonds with water. In contrast, NR is hydrophobic because its primary structure consists of a long-chain, nonpolar hydrocarbon known as polyisoprene. The DTG thermogram of the NR films shows that the maximum decomposition temperature of NR, referred to as NR-T_max_, is 390 °C.

The T_5%_, T_10%_, and T_50%_ values of the GG/NR films increased steadily with higher NR ratios. This trend suggests a decrease in the moisture content of the GG-based films and an increase in thermal stability resulting from the incorporation of NR. The char residue at 800 °C decreased as the NR ratio increased, attributed to NR’s lower char residue at 800 °C. The GG-T_max_ values for GG/NR films ranged from 294 to 296 °C, which is lower than the GG film’s value of 302 °C. This observation may be attributed to the presence of non-rubber components in fresh NR latex, which could diminish the interactions among GG molecules. The NR-T_max_ values for GG/NR films were between 386 and 389 °C, which is close to the NR film’s value of 390 °C.

### 3.3. Crystalline Structures

**Figure 4:**
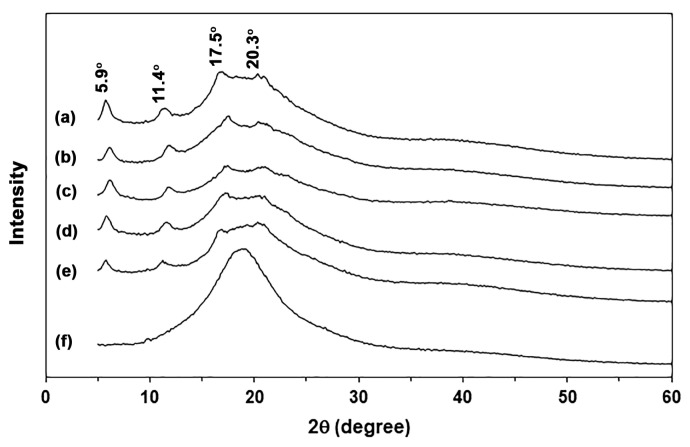
XRD patterns of GG/NR films with GG/NR ratios of (a) 100/0, (b) 95/5, (c) 90/10, (d) 80/20, (e) 60/40, and (f) 0/100 %*w*/*w*.

The crystalline structures of the film samples were examined using XRD patterns, as illustrated in Figure 4. The pure GG films exhibited XRD peaks at 2θ = 5.9°, 11.4°, 17.5°, and 20.3°. GG has been reported to have an XRD peak only at 2θ = 20.3° [45]. The additional XRD peaks identified in this work are likely indicative of the co-crystallization of GG in various other crystalline forms [20]. The XRD peaks observed at 2θ = 5.9° are indicative of B-type crystallinity, while the XRD peak at 2θ = 17.5° corresponds to A-type crystallinity in polysaccharides [50,51]. The presence of water, which acts as a de-structuring agent during compression molding, can enhance the mobility of GG chains. This process facilitates the optimization of their arrangement and packaging, ultimately resulting in the crystallization of GG [52]. The XRD results suggest that the presence of water, along with temperature and compression force, significantly influences the formation of GG crystallites.

There were no XRD peaks for the NR films, which means that the films were completely amorphous. The XRD peak positions of the GG/NR films precisely matched those of the pure GG films. This finding suggests that adding NR did not change the crystalline structures of the GG film matrix. The intensity of the XRD peaks for the GG matrix decreased as the ratio of NR increased. This trend is expected since a higher amount of NR dilutes the chain density of GG.

### 3.4. Phase Morphology

**Figure 5:**
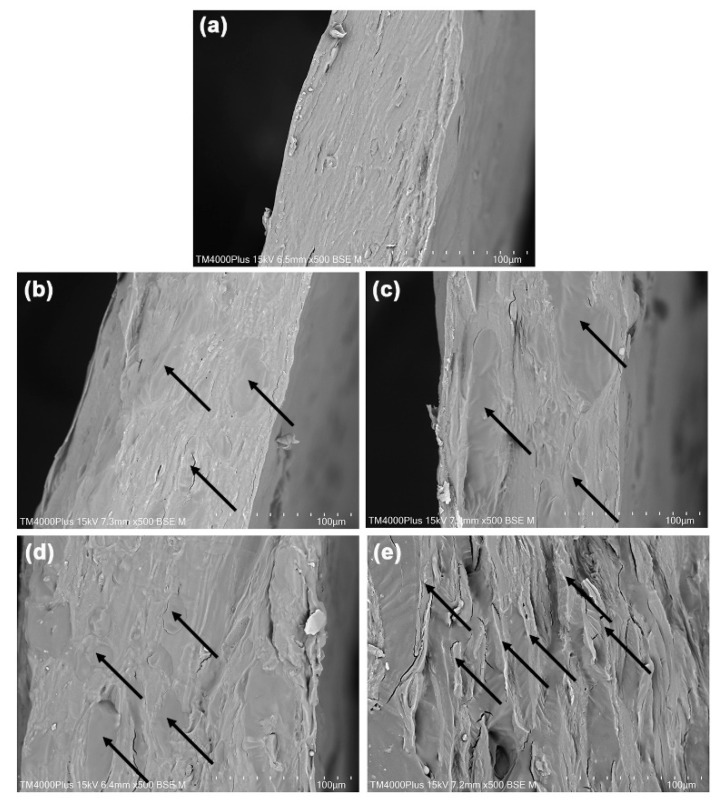
SEM images of cryo-fractured surfaces of GG/NR films with GG/NR ratios of (**a**) 100/0, (**b**) 95/5, (**c**) 90/10, (**d**) 80/20, and (**e**) 60/40 %*w*/*w*. Some NR phases are indicated by black arrows. All bar scales = 100 µm.

Figure 5 presents SEM images of the cryo-fractured surfaces of the film samples. The pure GG films shown in Figure 5a exhibit a dense film texture, with no visible GG particles. This observation suggests that water serves effectively as a de-structuring agent during the thermo-compression molding process of GG [20]. In the GG/NR films, the NR phases are identifiable within the GG film matrix, as indicated by the black arrows. The NR phases display smoother surfaces, while the GG phases are marked by rougher textures. The NR phases appear as dispersed particles that are evenly distributed throughout the GG/NR film matrix in samples containing 5%, 10%, and 20% NR. However, the GG/NR film with 40% NR shows NR phases that appear striped [see Figure 5e]. This change in appearance likely results from the increased NR content, which may have led to the agglomeration of NR particles.

**Figure 6:**
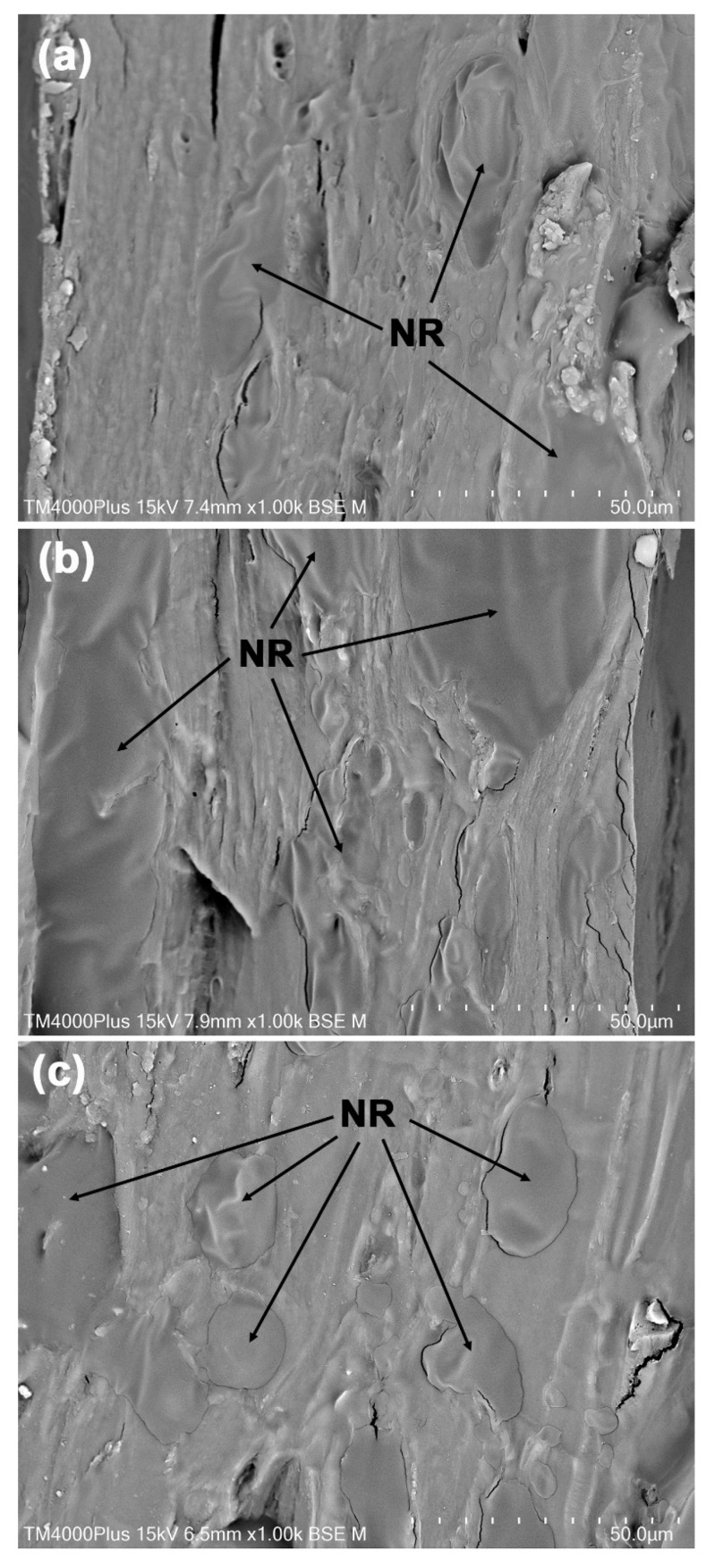
Expanded SEM images of cryo-fractured surfaces of GG/NR films with GG/NR ratios of (**a**) 95/5, (**b**) 90/10, and (**c**) 80/20 %*w*/*w*. All bar scales = 50 µm.

The GG and NR phases show a close bond, with no voids between them, indicating strong interfacial adhesion. This finding highlights the effective interfacial adhesion between the dispersed NR phases and the GG matrix. This finding is supported by the expanded SEM images shown in Figure 6, which specifically focus on GG-based films that contain 5%, 10%, and 20% NR. GG and NR are different in polarity. It is possible that the non-rubber substances that are present in fresh NR latex, particularly the proteins and phospholipids that are found on the surfaces of NR and lutoid particles [29], are responsible for the phenomenon of excellent interfacial adhesion in GG/NR films that was observed in this study. Proteins and phospholipids like these have the potential to function as phase compatibilizers for GG/NR films. These proteins and phospholipids improve interfacial adhesion between the hydrophilic GG phase and the hydrophobic NR phase because they are amphipathic molecules containing both hydrophilic and hydrophobic parts. This research is consistent with studies reporting the formation of hydrogen bonds between the functional groups of proteins on NR particle surfaces and the hydroxyl groups on carrageenan molecules [53].

### 3.5. Tensile Properties

**Figure 7:**
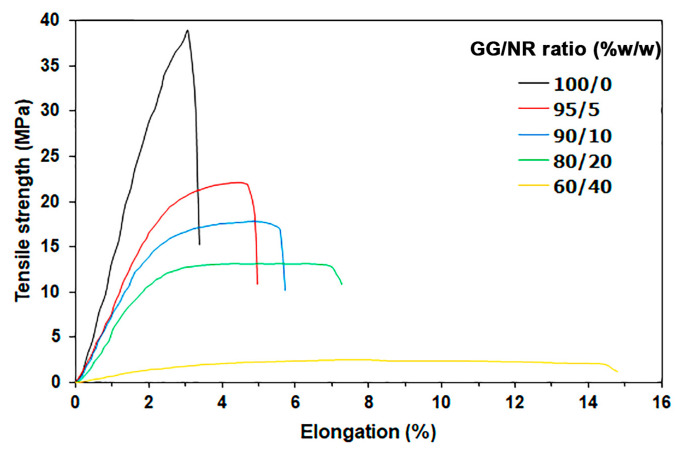
Tensile curves of GG/NR films with varying GG/NR ratios.

Figure 7 illustrates the tensile curves for the film samples, while the NR films are shown in Figure S1. The tensile data is summarized in Table 3. The pure GG films demonstrated a maximum tensile strength of 40.6 MPa, an elongation at break of 3.4%, and a Young’s modulus of 988.2 MPa. These results align with findings in the literature [54]. The GG film exhibited a maximum tensile strength of 40 MPa when it was prepared at a concentration of 12 g/L. This observation can be attributed to the significant entanglement of GG chains within films that are characterized by a high chain density.

The NR films demonstrated a maximum tensile strength of 0.4 MPa, an elongation at break of 787.4%, and a Young’s modulus of 0.9 MPa. The higher elongation at break indicates that NR films are much more flexible compared to pure GG films. The difference in flexibility between NR and GG films is attributed to their distinct glass transition temperatures (T_g_). At room temperature, NR remains in a rubbery state, with a T_g_ of approximately −65 °C [31]. In contrast, GG has a T_g_ around 108 °C [55], which means it remains in a glassy state at room temperature.

The blending of NR resulted in a decrease in the maximum tensile strength and Young’s modulus of the GG film while simultaneously enhancing its elongation at break. The elongation at break increased by 41% for a 5% NR blend, 65% for a 10% NR blend, 115% for a 20% NR blend, and 415% for a 40% NR blend. This discovery indicates that blending NR enhances the flexibility of GG-based films. This versatile NR has been utilized to increase the flexibility of stiffer polymers [32,33,34,35]. For these blends to achieve greater flexibility when combined with NR, there must be adequate phase compatibility between the polymer matrix and the dispersed NR phases [33,35]. The SEM analysis shows that GG/NR films have strong adhesion at the interface, which means that the phases are compatible. Proteins and phospholipids in fresh NR latex can act as effective phase compatibilizers for GG/NR films. They reside at the interfaces between the GG and NR phases, increasing interfacial adhesion and enhancing the flexibility of GG-based films. This supports the tensile test results, which indicate that the NR blend makes GG-based films more flexible. The tensile strength and tensile modulus of the GG-based films with 5% and 10% NR, developed in this study, were similar to those of conventional polyethylene films used for packaging. Typically, polyethylene films exhibit a tensile strength of 20 MPa and a tensile modulus of 590 MPa [54]. The mechanical properties of the GG/NR films suggest their potential for packaging applications, particularly in scenarios where strength and stiffness are required, similar to those provided by conventional polyethylene films.

### 3.6. Water Resistance

**Figure 8:**
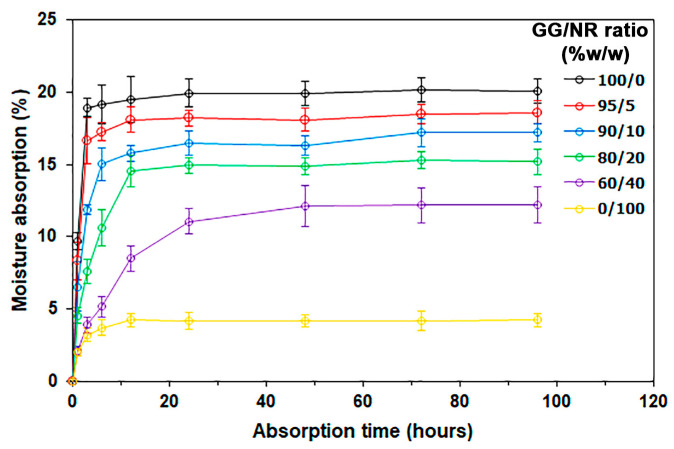
Moisture absorption of GG/NR films with varying GG/NR ratios.

The water resistance of the GG/NR films was investigated from their moisture absorption, water dissolution, surface wettability, and water vapor permeability. Figure 8 presents the moisture absorption curves of the film samples that were tested in a desiccator maintained at a relative humidity of 75%. The pure GG films exhibited the highest level of moisture absorption, reaching a constant value of about 20% after 3 h. This observation can be attributed to the high content of –OH groups present in the molecular structure of the GG [56]. The NR films demonstrated a stable moisture absorption value of about 4% after 12 h. This finding indicates that NR films possess a high water-resistant property due to the hydrophobic nature of *cis*-1,4-polyisoprene molecules, which is the main component of NR films [29]. The moisture absorption value of GG/NR films was lower than that of pure GG films, and this value consistently decreased as the ratio of NR increased. The GG/NR films with NR ratios of 5%, 10%, 20%, and 40% exhibited moisture absorption values at 96 h of 19%, 17%, 15%, and 12%, respectively. The results indicate that blending NR with GG films reduced moisture absorption, which can be attributed to the hydrophobic nature of the dispersed NR phases.

The water dissolution of the film samples was evaluated over a 24 h period while shaking at 25 °C. The results are presented in Table 4. The pure GG films had the highest value of water dissolution at 57.74%, and the NR films had the lowest value at 12.71%. The water-soluble fractions of the NR films are presumed to consist of non-rubber components that can dissolve in water, such as carbohydrates, proteins, phospholipids, and inorganic salts [57]. For GG/NR films, the water dissolution value decreased with an increasing ratio of NR. This finding shows that GG/NR films exhibit greater water resistance compared to pure GG films.

**Figure 9:**
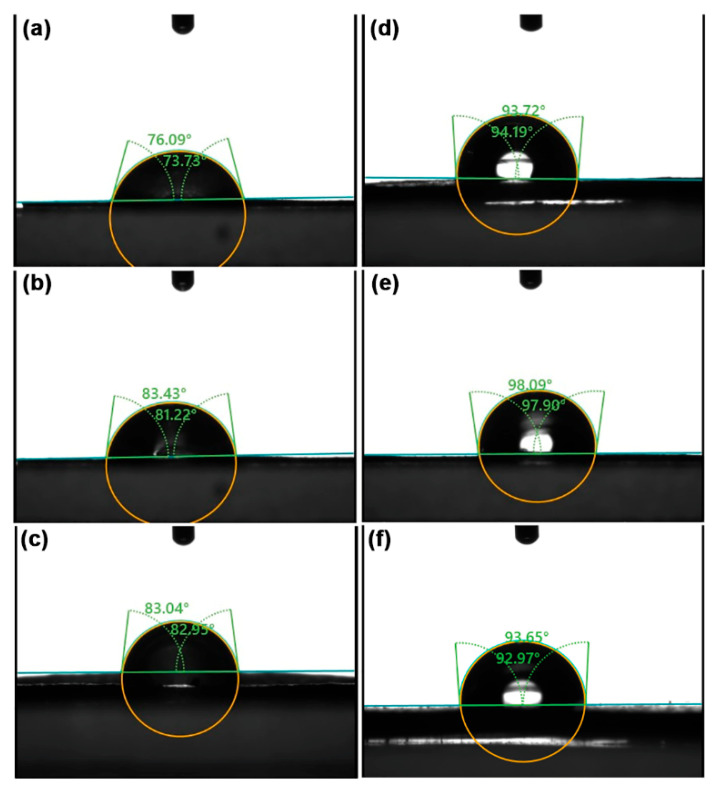
Water contact angles of GG/NR films with GG/NR ratios of (**a**) 100/0, (**b**) 95/5, (**c**) 90/10, (**d**) 80/20, (**e**) 60/40, and (**f**) 0/100 %*w*/*w*.

The surface wettability of the film samples was assessed by measuring the water contact angles, as illustrated in Figure 9, with the results summarized in Table 4. Films with higher water contact angles indicate that they have lower surface wettability or greater water resistance. The pure GG and NR films exhibit water contact angles of 75.84° and 94.26°, respectively. This data indicates that pure GG films possess higher surface wettability compared to NR films. An increased NR ratio raised the water contact angles of the GG/NR films. These findings can be attributed to the highly hydrophobic nature of the rubber component, which has low surface energy and minimal intermolecular interaction with water that is dispersed within the GG film matrix. This property leads to an increased contact angle, which signifies decreased surface wettability in the GG/NR films [58].

Water vapor permeability (WVP) is a crucial factor in food packaging applications because it controls the evaporation of moisture from food and helps preserve its freshness. Table 4 shows that pure GG films had the highest WVP value of 10.15 × 10^−10^ g·mm^−1^·h^−1^·Pa^−1^. The NR films showed the lowest WVP value, which is 1.92 × 10^−10^ g·mm^−1^·h^−1^·Pa^−1^. The WVP of the blend films significantly decreased with an increase in the content of NR. These experimental results demonstrate that the addition of NR enhances the water resistance of GG-based films. The experimental findings related to moisture absorption, water solubility, surface wettability, and water vapor permeability suggest that incorporating NR improves the water-resistant properties of GG films. Consequently, this improvement may enable the application of these GG-based films in a broader and more suitable range of food packaging applications, for example, dry foods and short shelf-life products.

### 3.7. Film Opacity and Color

**Figure 10:**
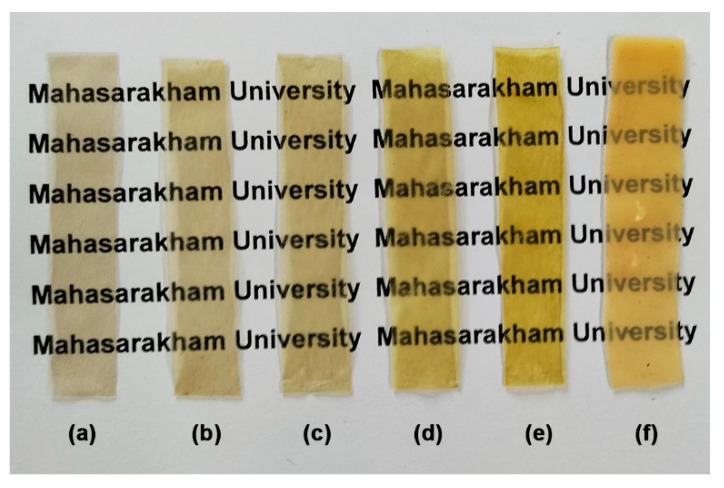
Visible transparency of GG/NR films with GG/NR ratios of (**a**) 100/0, (**b**) 95/5, (**c**) 90/10, (**d**) 80/20, (**e**) 60/40, and (**f**) 0/100 %*w*/*w*.

Table 5 reports the effect of blending NR on the thickness, opacity, and color parameters of the GG-based films. The thicknesses of the GG/NR films tended to increase as the NR ratio increased. A higher NR ratio in the blended pellets increases melt viscosity during the thermo-compression process, which may explain this finding. The film opacity of GG/NR films increased as the NR ratio increased because the film opacity of NR films (3.55 mm^−1^) was higher than that of pure GG films (0.77 mm^−1^). However, all the GG/NR films were still transparent, and the letters on the back could be seen, as shown in Figure 10.

The results of color parameters indicated that the lightness (L* value, ranging from black 0 to white 100) of pure GG, GG/NR, and NR films was similar. The lightness values ranged from 40.61 to 42.42. Consequently, blending NR with GG did not have a significant effect on the lightness of the GG films. In the color parameter a* (ranging from red at +120 to green at −120), the color of the GG/NR films shifts slightly toward the greener zone. In the color parameter b* (ranging from yellow at +120 to blue at −120), the addition of NR caused a significant shift in the b* values toward the yellower zone due to non-rubber substances in the fresh NR latex [59]. This observation aligns with the film color depicted in Figure 10, which shows that the film becomes increasingly yellow as the NR ratio increases. However, for NR films, the b* value was found to be low (5.03). NR films have an obvious yellow color after drying. Nevertheless, when maintained at ambient temperature and humidity for 14 days, NR films exhibit increased opacity. The decrease in b* value for the pure NR films may reflect conditioning-induced changes. While moisture absorption may play a role, alternative mechanisms, such as bleaching or the migration of non-rubber components within the NR film, could also contribute.

### 3.8. Biodegradation in Soil

**Figure 11:**
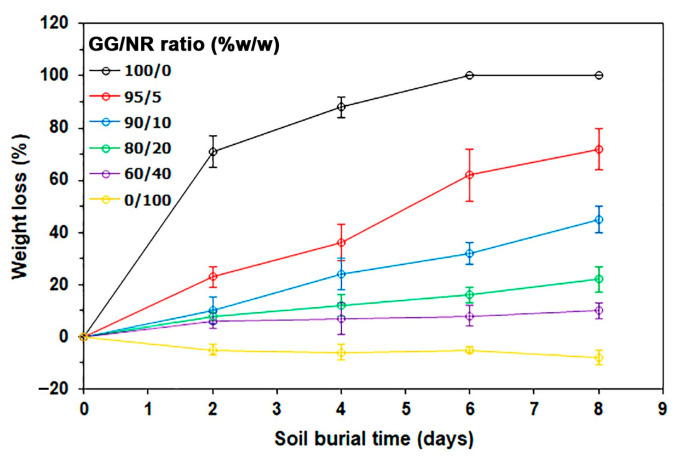
Weight losses of GG/NR films for soil burial test.

Figure 11 illustrates the weight reduction in sample films subsequent to the soil burial test. The findings indicate that pure GG films undergo total degradation within 6 days of burial, leading to a 100% loss in weight due to their high hydrophilicity. The weight loss in GG/NR films diminishes with an increase in NR content. This phenomenon is probably attributable to the hydrophobic properties of the NR phases, which impede the infiltration of water molecules into the GG matrix. Moreover, NR films demonstrating negative weight loss may be ascribed to the NR’s lack of degradation during the testing period, with soil adhering to the film surface, leading to a weight exceeding the initial film weight post-burial.

**Figure 12:**
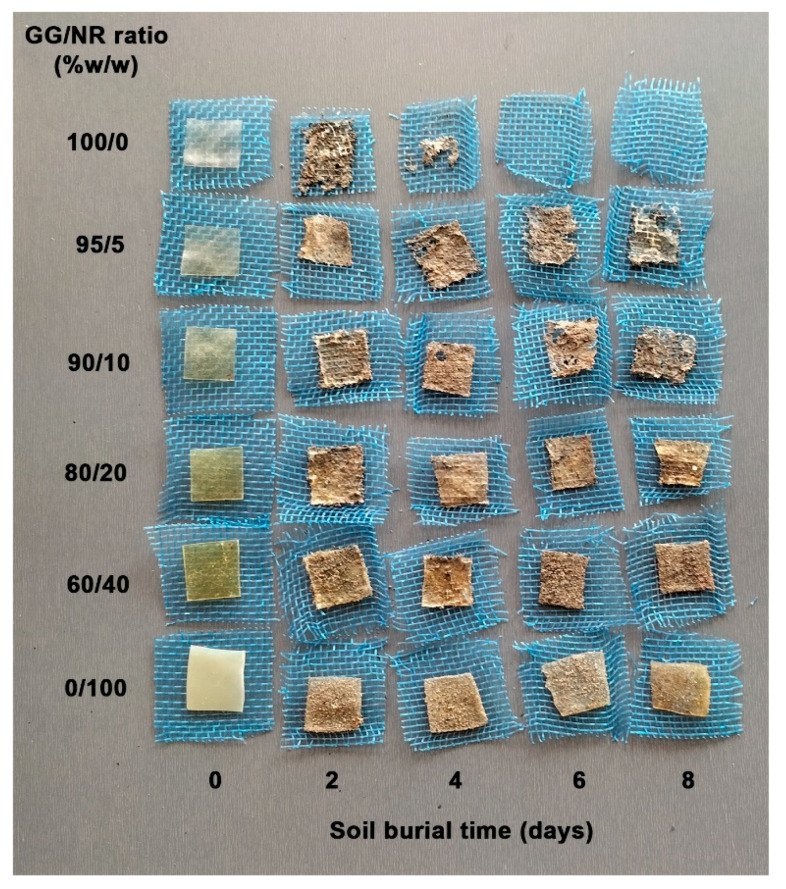
Visible characteristics of GG/NR films before (0 day) and after burial in soil for 2, 4, 6, and 8 days.

The study of the biodegradation behaviors of polysaccharide-based materials during soil burial tests can be evaluated by monitoring the change in the deterioration of the sample films [60,61]. Figure 12 shows photographs of film samples taken before and after biodegradation by soil burial tests. During the eight-day experiment, the film samples were buried in soil for different lengths of time. The pure GG film exhibited notable deterioration after only two days of being buried. It was completely biodegraded at the end of the six-day test, presumably due to the actions of mannan-degrading enzymes, including β-mannanases, β-mannosidases, α-galactosidases, and acetyl-mannan esterases [62]. These enzymes are found in several groups of soil microorganisms whose primary function is the decomposition of plant material, including fungi such as *Aspergillus* and *Trichoderma*, and bacteria such as *Bacillus*, *Cellulomonas*, and *Streptomyces* [63].

The NR films exhibited no noticeable deterioration after being buried for 8 days, likely due to their high hydrophobicity. In contrast, GG/NR films exhibited signs of deterioration after burial; however, the extent of this deterioration was significantly less than that observed in pure GG films. This observation aligns with findings on the soil degradation of starch/NR blended films, which demonstrated slower degradation rates as the NR ratio increased [64]. The findings of these experiments demonstrate that GG/NR blends are biodegradable materials that are suitable for utilization in environmentally friendly packaging applications.

In addition, the findings suggest that NR blends result in slower deterioration and degradation. This conclusion is supported by the experimental data on water-resistant properties of GG/NR films, all of which decrease as the NR ratio increases. The polymers experience a slower rate of biodegradation as a result of the increased water-resistant properties [65,66]. In this study, the GG/NR films were buried for the same amount of time; their biodegradation rate decreased as the NR ratio increased. The formulated GG/NR blends are potentially viable substitutes for sustainable, single-use, or disposable products. This study improves our comprehension of biodegradability behavior, which is essential for the effective management of biodegradation in diverse packaging applications.

## 4. Conclusions

Guar gum/natural rubber (GG/NR) films were created by hand-kneading GG powder and fresh NR latex prior to thermo-compression. The films had GG/NR ratios of 100/0, 95/5, 90/10, 80/20, and 60/40 %*w*/*w*. SEM analysis revealed strong interfacial interactions between the dispersed NR phases and GG matrix, as corroborated by FTIR analysis. The GG/NR films exhibited a decrease in maximum tensile strength and Young’s modulus, accompanied by an increase in elongation at break as the NR ratios increased. Specifically, there were increases in elongation at break of 41%, 65%, 115%, and 415% for NR ratios of 5%, 10%, 20%, and 40%, respectively, when compared to the pure GG films. The water-resistant properties of GG-based films—such as moisture absorption, water dissolution, water contact angle, and water vapor permeability—improved with higher NR ratios. Additionally, the incorporation of NR reduced the degradation rate of GG-based films during soil burial. This research represents a significant advancement in biomaterial development by producing GG-based materials that enhance both flexibility and water resistance. Integrating dispersed NR into the GG matrix without the use of additional chemicals achieves this improvement. These GG/NR films are sustainable materials that have the potential to replace petroleum-based products in single-use packaging applications, particularly due to their biodegradability and renewable resource origin, positioning them as an environmentally friendly alternative.

## Figures and Tables

**Table 1:** Formulations of GG/NR pellets.

GG/NR Ratio (%*w*/*w*)	GG (g)	Fresh NR Latex (g)	NR in Fresh NR Latex (g) ^1^	Water in Fresh NR Latex (g) ^2^	Distilled Water (g)
100/0	10.00	-	-	-	30.00
95/5	10.00	1.54	0.54	0.76	29.24
90/10	10.00	3.20	1.12	1.56	28.44
80/20	10.00	7.14	2.50	3.50	26.50
60/40	10.00	19.02	6.66	9.32	20.68

^1^ Calculated based on 35 wt% DRC of fresh NR latex. ^2^ Calculated based on 49 wt% TSC of fresh NR latex.

**Table 2:** Thermal decomposition properties of GG/NR films.

GG/NR Ratio (%*w*/*w*)	T_5%_ (°C) ^1^	T_10%_ (°C) ^1^	T_50%_ (°C) ^1^	Char Residue at 800 °C (%) ^1^	GG-T_max_ (°C) ^2^	NR-T_max_ (°C) ^2^
100/0	81	121	309	18.05	302	-
95/5	92	148	311	17.29	296	386
90/10	107	175	323	15.30	295	387
80/20	118	197	352	14.65	296	386
60/40	143	244	373	11.38	294	389
100/0	322	351	389	2.73	-	390

^1^ Obtained from TG thermograms. ^2^ Obtained from DTG thermograms.

**Table 3:** Tensile properties of GG/NR films.

GG/NR Ratio (%*w*/*w*)	Maximum Tensile Strength (MPa)	Elongation at Break (%)	Young’s Modulus (MPa)
100/0	40.6 ± 2.5 ^f^	3.4 ± 0.5 ^a^	988.2 ± 15.6 ^f^
95/5	23.1 ± 1.4 ^e^	4.8 ± 0.8 ^b^	539.9 ± 12.6 ^e^
90/10	18.8 ± 1.9 ^d^	5.6 ± 0.6 ^b^	437.4 ± 17.2 ^d^
80/20	13.9 ± 1.2 ^c^	7.3 ± 1.4 ^c^	328.8 ± 16.8 ^c^
60/40	3.2 ± 0.6 ^b^	17.5 ± 1.5 ^d^	50.9 ± 8.4 ^b^
100/0	0.4 ± 0.1 ^a^	787.4 ± 10.7 ^e^	0.9 ± 0.2 ^a^

Values are shown as mean ± standard deviation (*n* = 3). Column values represented by the letters (a, b, c, d, e, and f) demonstrate substantial differences (*p* < 0.05).

**Table 4:** Water dissolution, water contact angle, and water vapor permeability of GG/NR films.

GG/NR Ratio (%*w*/*w*)	Water Dissolution (%)	Water Contact Angle (°)	Water Vapor Permeability (×10^−10^ g·mm^−1^·h^−1^·Pa^−1^)
100/0	57.74 ± 2.87 ^e^	75.84 ± 2.54 ^a^	10.15 ± 1.14 ^e^
95/5	56.18 ± 3.56 ^e^	82.52 ± 3.12 ^b^	7.59 ± 0.24 ^d^
90/10	51.32 ± 3.24 ^d^	82.95 ± 2.76 ^b^	6.07 ± 0.17 ^c^
80/20	46.96 ± 4.12 ^c^	93.87 ± 3.11 ^c^	4.50 ± 0.31 ^b^
60/40	31.15 ± 4.84 ^b^	97.89 ± 3.52 ^d^	2.16 ± 0.24 ^a^
100/0	12.71 ± 1.25 ^a^	94.26 ± 4.28 ^c^	1.92 ± 0.15 ^a^

Values are shown as mean ± standard deviation (*n* = 3). Column values represented by the letters (a, b, c, d, and e) demonstrate substantial differences (*p* < 0.05).

**Table 5:** Thickness, opacity, and color parameters of GG/NR films.

GG/NR Ratio (%*w*/*w*)	Film Thickness (mm)	Film Opacity (mm^−1^)	Color (CIELAB Space)		
			L*	a*	b*
100/0	0.14 ± 0.06 ^a^	1.45 ± 0.03 ^a^	41.18 ± 0.03 ^a^	−0.44 ± 0.04 ^f^	4.44 ± 0.33 ^a^
95/5	0.18 ± 0.04 ^a^	1.57 ± 0.03 ^b^	40.88 ± 0.08 ^a^	−1.31 ± 0.02 ^e^	8.09 ± 0.06 ^b^
90/10	0.21 ± 0.05 ^a,b^	1.60 ± 0.05 ^b^	40.61 ± 0.06 ^a^	−2.16 ± 0.04 ^c^	10.57 ± 0.30 ^c^
80/20	0.29 ± 0.04 ^c^	1.57 ± 0.04 ^b^	41.73 ± 0.35 ^a^	−2.99 ± 0.10 ^b^	15.33 ± 0.36 ^d^
60/40	0.42 ± 0.03 ^d^	1.58 ± 0.05 ^b^	40.67 ± 0.62 ^a^	−3.29 ± 0.24 ^a^	18.21 ± 0.71 ^e^
100/0	0.44 ± 0.02 ^d^	2.55 ± 0.08 ^c^	42.42 ± 0.47 ^b^	−1.75 ± 0.09 ^d^	5.03 ± 0.26 ^a^

Values are shown as mean ± standard deviation (*n* = 3). Column values represented by the letters (a, b, c, d, e, and f) demonstrate substantial differences (*p* < 0.05).

## Data Availability

The original contributions presented in this study are included in the article/Supplementary Materials. Further inquiries can be directed to the corresponding authors.

## Supplementary Materials

The following supporting information can be downloaded at https://www.mdpi.com/article/10.3390/polym18080956/s1, Figure S1: Tensile curve of NR film.
