# Supplementary material for: Improving the Flexibility and Water Resistance of Thermo-Compressed Guar Gum Films by Blending Natural Rubber for Use in Sustainable Packaging Applications
Source: Polymers (Basel). 2026 Apr 14;18(8):956. doi: 10.3390/polym18080956 (PMC13120185; doi:10.3390/polym18080956)
Supplement: Supplementary file 1 [file polymers-18-00956-s001.zip › polymers-4243080-supplementary.pdf]

Supplementary Material

Improving the Flexibility and Water Resistance of Thermo-Compressed Guar Gum Films by Blending Natural Rubber for Use in Sustainable Packaging Applications

Prasong Srihanam [1,*], Nuanchai Khotsaeng [2] and Yodthong Baimark [1,*]

[1] Biodegradable Polymers Research Unit, Department of Chemistry and Centre of Excellence for Innovation in Chemistry, Faculty of Science, Mahasarakham University, Maha Sarakham 44150, Thailand
[2] Faculty of Science and Health Technology, Kalasin University, Namon District, Kalasin 46230, Thailand; nuanchai.k@ksu.ac.th
* Correspondence: prasong.s@msu.ac.th (P.S.); yodthong.b@msu.ac.th (Y.B.)

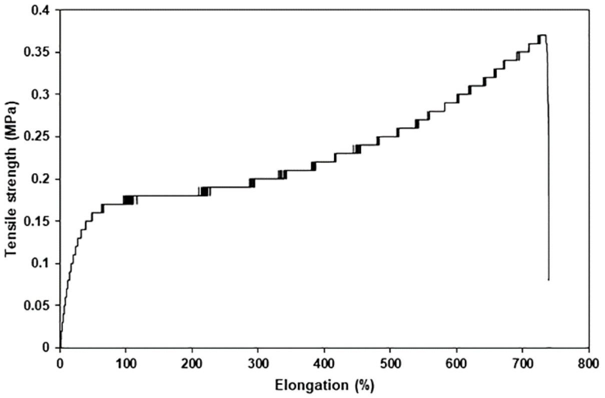

Figure S1. Tensile curve of NR film.
